# Identification of Small-Molecule Inhibitors of the HuR/RNA Interaction Using a Fluorescence Polarization Screening Assay Followed by NMR Validation

**DOI:** 10.1371/journal.pone.0138780

**Published:** 2015-09-21

**Authors:** Zhonghua Wang, Akash Bhattacharya, Dmitri N. Ivanov

**Affiliations:** Department of Biochemistry, University of Texas Health Science Center at San Antonio, San Antonio, TX, 78229, United States of America; Tsinghua University, CHINA

## Abstract

The human antigen R (HuR) stabilizes many mRNAs of proto-oncogene, transcription factors, cytokines and growth factors by recognizing AU-rich elements (AREs) presented in their 3’ or 5’ untranslated region (UTR). Multiple lines of experimental evidence suggest that this process plays a key role in cancer development. Thus, destabilizing HuR/RNA interaction by small molecules presents an opportunity for cancer treatment/prevention. Here we present an integrated approach to identify inhibitors of HuR/RNA interaction using a combination of fluorescence-based and NMR-based high throughput screening (HTS). The HTS assay with fluorescence polarization readout and Z’-score of 0.8 was used to perform a screen of the NCI diversity set V library in a 384 well plate format. An NMR-based assay with saturation transfer difference (STD) detection was used for hits validation. Protein NMR spectroscopy was used to demonstrate that some hit compounds disrupt formation of HuR oligomer, whereas others block RNA binding. Thus, our integrated high throughput approach provides a new avenue for identification of small molecules targeting HuR/RNA interaction.

## Introduction

Post-transcriptional regulation of gene expression is a general theme in all living organisms [1]. RNA binding proteins (RBPs) that associate with specific mRNA and function as mRNA turnover and translation regulatory RNA binding protein (TTR-RBP) have emerged as pivotal post-transcriptional regulators of gene expression in mammalian cells [2–4]. HuR stabilizes many mRNAs by recognizing AU-rich elements (AREs), which are presented in 3’ or 5’ untranslated region (UTR) of many mRNAs, encoding proto-oncogenes, transcription factors, cytokines, and growth factors [5–17]. HuR contains three RNA recognition motifs (RRMs): N-terminal tandem RRM1-2 and C-terminal RRM3 [18]. Although the sequence alignment indicates that all three RRMs have the same canonical β_1_α_1_β_2_β_3_α_2_β_4_ fold [19,20], the function of the individual RRM domains is different. RRM1 and RRM2 are mainly responsible for binding to AREs [21,22], whereas RRM3 binds to the polyA tail and other proteins [21,23]. RRM3 does not contribute to high affinity recognition but instead it is required for cooperative assembly of HuR oligomers when ARE substrates are at least 18 nucleotides in length [24].

Elevated expression of HuR is linked to carcinogenesis in many human tumors and correlates with poor outcome [25–29]. For example, the over-expression of HuR in brain cancers promotes the growth of brain tumor such as Glioblastoma multifome and medullobastoma [30,31]. The overexpression also correlates with resistance to chemotherapeutic agents in a variety of cancers such as brain cancer [16], and breast cancer [32]. The knockdown of HuR increased sensitivity to chemotherapeutic drugs [16,33] and promotes apoptosis [34]. HuR has been studied for decades since its discovery in 1996 [18]. It has merged as a promising drug target for cancer treatment/prevention. Our goal is to develop an integrated approach for identification of specific HuR inhibitors which destabilize HuR/RNA interaction. We utilize an assay with florescence polarization (FP) readout, which is a homogeneous method for rapid and quantitative analysis of molecular interactions [35], as our primary HTS assay. The assays yielded the Z’score of 0.8, indicating robustness and suitability for HTS. Here, we also take advantage of modern NMR techniques and use ligand-based and protein-based approaches as secondary assays for validation of the hits from primary screen. [36–42]. The advantage of the saturation transfer difference (STD-NMR) is that it can be used to observe binding to large proteins [43–45], whereas the chemical shift perturbation using 2D ^1^H-^15^N HSQC is unparalleled in its ability to identify the binding site, even though its utility is limited to smaller proteins that yield good NMR spectra [45]. 12 compounds identified in the primary screen of the NCI diversity V library were further analyzed by STD-NMR. 4 of 12 compounds were shown to interact with HuR directly. The data from ^1^H-^15^N protein HSQC spectra revealed that one of the compounds (C10) disrupts HuR/RNA interaction, whereas another compound (C11) impedes HuR oligomerization. Our results suggest that this integrated approach can be a valuable strategy for screening compound libraries for inhibitors of the HuR function.

## Material and Methods

### RNA sample

5’-FAM labeled RNA oligonucleotide (AUUUUUAUUUU) derived from the 3’ UTR of the c-Fos proto-oncogene was purchased from MWG operon (HPLC purified and RNase free). Unlabeled c-Fos RNA oligo was purchased from IDT (HPLC purified and RNase free).

### Protein production

Full length (residue 1–326), RRM1-2 (residue 1–190), RRM1 (residue 1–98) and RRM2 (residue 102–186) constructs of HuR was PCR amplified from pMal-c2x plasmid containing full length HuR (gift from Dr. Gorospe) and cloned into pET14b vector. They were both expressed in *E*.*coli*. BL21 Rosetta cells. The culture was grown to OD_260_~0.6 at 37°C, and the protein expression was induced by addition of 1mM IPTG. The incubator temperature was reduced to 18°C immediately following IPTG addition and the cells cultured for additional 16–18hrs. The cells were harvested by centrifugation for 20 min at 6000rpm and sonicated in the presence of protease inhibitors and 15% Glycerol in 50mM Tris pH8.0. Cell lysate was spun down at 18000rpm and the supernatant was collected. The protein was purified using metal chelation chromatography, followed by size exclusion chromatography on the G75 (for the RRM1-2, RRM1 and RRM3 constructs) or G200 (for full-length construct) resin. Size exclusion chromatography buffer contained 50mM phosphate buffer, 100mM NaCl, 5mM TCEP at pH7.0. ^13^C/^15^N or ^15^N labeled proteins were expressed in ^13^C/^15^N or ^15^N-enriched M9 minimal media respectively and purified using the same protocol.

### Fluorescence polarization (FP) assay

Fluorescence polarization measurements were performed in the 384-well plate format on a Biotek Synergy2 plate reader using 480/20nm excitation and 520/20nm emission filters. The NCI diversity set V library containing 1597 compounds was screened in this study. All samples were dissolved in phosphate buffer (50mM Napi, and 100mM NaCl, 5mM TCEP at pH 7.0). The assay mixture contained 50nM HuR and 10nM RNA to yield FP values approximately 80% of the maximum value observed for fully bound RNA. NCI diversity set V library in 96-well format (10mM stock in DMSO) was transferred to 384-well plate, and then diluted into 50uM in phosphate buffer. 5ul of 50uM compound stock was added to 20 ul assay mixture to yield 10uM final compound concentration.

### NMR spectroscopy and data collection and assigment

Protein samples were prepared in 50mM phosphate buffer at pH 7.0 containing 100mM NaCl, 5mM TCEP in D_2_O or H_2_O. Saturation transfer difference (STD) NMR spectra were acquired at 298K on Avance III Bruker 500 MHz spectrometer equipped with a 1.7mm TCI cryogenic probe and an automated SampleJet sample changer. Compound concentration in the samples was 0.5 mM and the HuR concentration was 10uM. Protein saturation was achieved using the STDDIFFESGP.3 pulse. The on resonance was set for -500Hz (-1ppm) and off resonance was set for 25000Hz (50ppm), the saturation time is 3s. 16 scans were acquired with 1.5s relaxation delay between the scans. The data was processed using nmrPipe and plotted using Xmgrace. 2D ^1^H-^15^N HSQC was acquired with 2048 and 128 complex points in the direct and indirect dimensions, respectively. Protein concentration was 0.1~0.2mM. Backbone assignment of RRM1-2 was derived from the analysis of 3D HNCACB, CBCACONH.

### Analytical ultracentrifugation (AUC)

Sedimentation velocity analytical ultracentrifugation was performed on a Beckman XLA analytical ultracentrifuge using an AN50 Ti rotor with standard Epon 2-channel centerpieces. The samples were spun at 25000rpm for~12hrs at 20 C. 25 scans measuring absorbance at 280nm were collected. The data were analyzed using Ultrascan III software.

## Results

### FP-based High throughput screening using NCI diversity set V library

**Fig 1:**
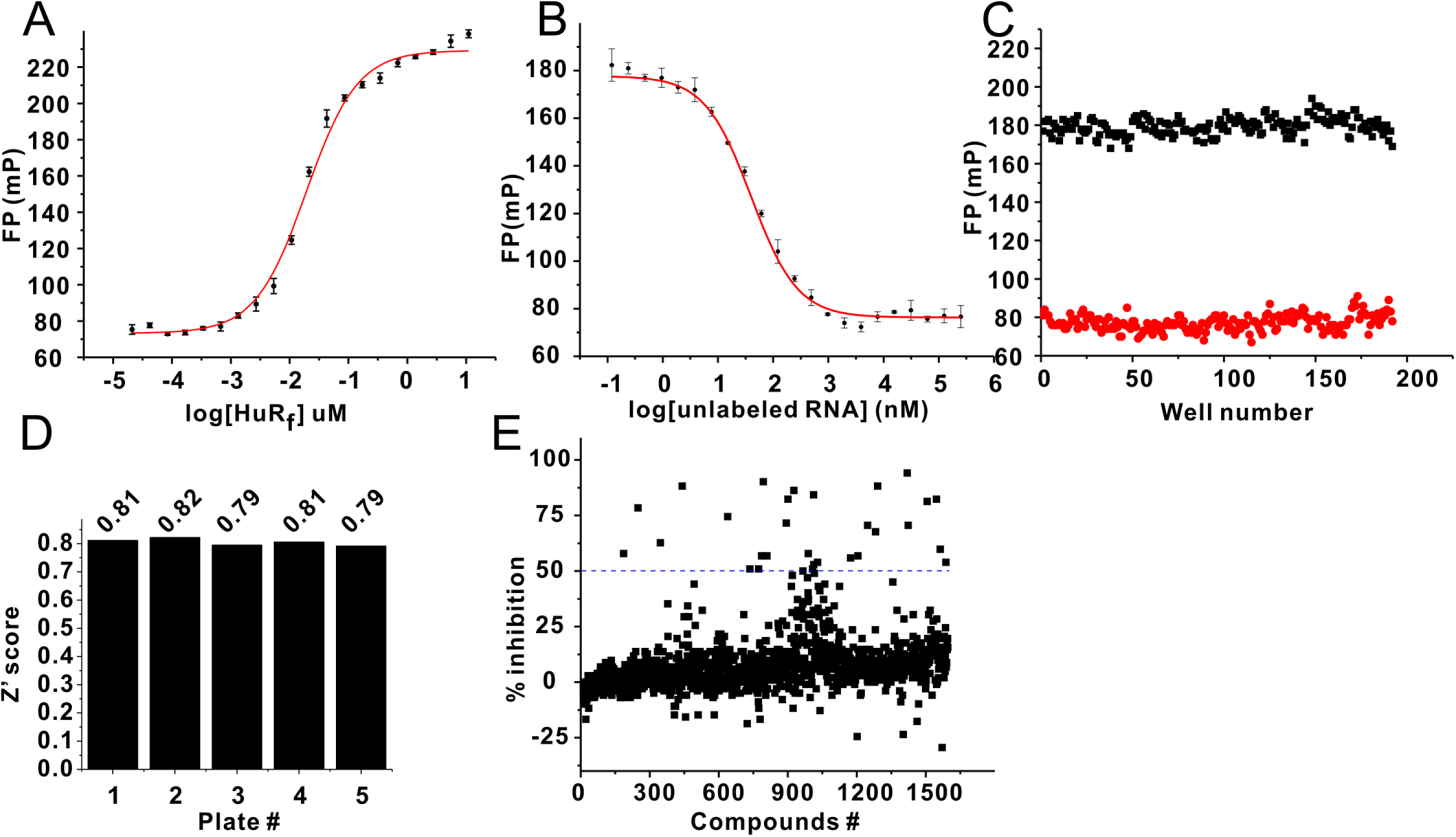
HTS screening using NCI diversity set V compounds library. (A) Affinity of C-fos to Full length HuR. The Kd was derived by fitting to the equation of ΔP = ΔPmax*[HuR]/(Kd+[HuR])[46]. (B) Competition assay using unlabeled C-fos RNA. IC50s were obtained by fitting to the equation of ΔP = ΔPmax/(1+10^((Logx_0_-X)*p)). (C) Z’score of HTS, calculated using Z’ = 1-[3(σ_p_+σ_n_)/|μ_p_-μ_n_|] [47], where μ, σ are the means, standard deviation respectively. p, n are positive control and negative control respectively. The bottom (red) is positive control measured in the presence of 10uM unlabeled RNA in a total volume of 25ul. The top (black) is negative control measured in the presence of 0.5% DMSO at 10nM RNA and 50nM HuR used in the screen. (D) Z’ score from each plate under screening condition. (E) Percentage of inhibition from primary screening using Diversity V library. >50% inhibition was used as a threshold to pick initial hits (blue dotted line).

We first characterized the binding of the full-length HuR with 11-mer polyribonucleotide derived from the 3’ UTR of the mRNA of the C-fos oncogene (Fig 1A). The binding curves were obtained by keeping the 5’FAM-RNA at 10 nM and adding increasing amounts of HuR. The apparent dissociation constant for the HuR/RNA binding was estimated to be approximately 20 nM (Fig 1A) using the simplified equation as described [46]. To evaluate the expected effect of the inhibitors on the FP readout of the essay, we measured displacement of the fluorescently labeled RNA by the unlabeled RNA (Fig 1B). In displacement assay, the 10nM of 5’FAM-RNA concentration was mixed with 50nM at which fluorescence polarization value was approximately 80% of the maximal value. Fluorescence polarization was measured as a function of the unlabeled RNA concentration. IC50 was obtained (~40nM) by fitting to the equation specified in the figure legend (Fig 1B)

To evaluate the assay readiness, we performed competition assay with unlabeled RNA. For FP based competitive HTS, Z’ score is the quality factor for the evaluation of an assay: the higher of Z’ value, the more robust of the assay is [48]. We calculated Z’ score as of 0.75 using the established protocol [47]. To evaluate the quality of HTS, we also calculated Z’ score for each plate under screening condition (Fig 1D). The average Z’ score from different plates is about 0.80. These results indicate our FP assay is reproducible and reliable HTS (Fig 1C and 1D).

We then performed initial screening using NCI diversity set V, containing 1597 compounds as described in the Material and Methods section. The standard deviation for FP measurement in the screen was 18.2 in unfiltered raw data (S1 Fig). Compounds producing FP readings significantly higher than the average (2σ) were ignored as higher FP readings are usually produced by compounds causing protein precipitation and/or fluorescence quenching. We also discard the hits which produce a decrease in FP values if the total fluorescence reading is 5-standard deviation below the average. The compounds falling into that regime typically have intrinsic fluorescence. The standard deviation for FP after removing 18 compounds was 12.4 with the average of fluorescence reading at 171.40 (S1 Fig). Compounds with the FP change of 3-standard deviation below the average FP value (S1 Fig) and above 50% inhibition (Fig 1D) were selected for subsequent hit verification. 30 of 1597 compounds were picked from our primary screening for retesting in original FP screening assay (S1 Fig). 16 compounds were selected using the same cutoff as the initial hits pick-up (3-standard deviation below the average FP value) (S1 Fig). To further verify the hits before running STD-NMR assay, we performed the analysis of dose responses on the selected hits (S1 Table and S2 Fig). The compounds produced higher FP values at higher concentration of compounds were further removed. Finally, 12 out of 1597 (hits rate is ~0.75%) compounds were selected for secondary screening using STD-NMR.

### Secondary screening using Saturation transfer difference (STD)

**Fig 2:**
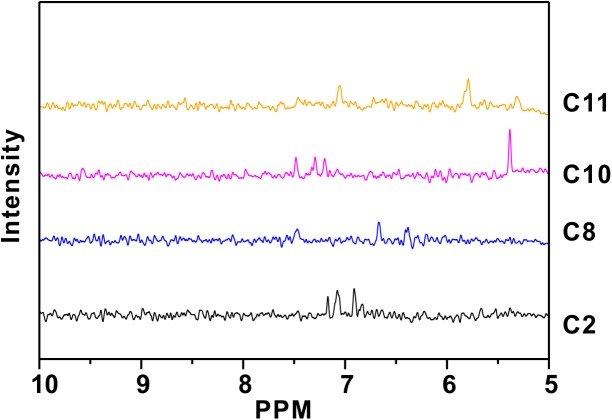
Secondary screening as hits validation using NMR STD approach. 4 out of 12 compounds from our primary screening showed direct interactions with HuR. The data were processed and plotted using the shell script developed for automation in our lab, making STD screening faster, robust and competitive to HTS.

NMR-based assays are good secondary assays for HTS, because they provide evidence for direct interaction of the hit compounds with the target protein, thus removing the possibility of the interaction with the RNA or another false-positive effect arising from the direct influence of the hit compound on the fluorescent tag. STD-NMR is a powerful way of monitoring small-molecule/protein interaction because of the ease to implement, the need for low concentration of protein (less than 10uM protein), and the no-limitation for molecular weight. 4 out of 12 compounds from the primary screens displayed STD difference effects (Fig 2) and those compounds seem to have distinct structural features (S3 Fig).

### ^1^H-^15^N HSQC as secondary screening

**Fig 3:**
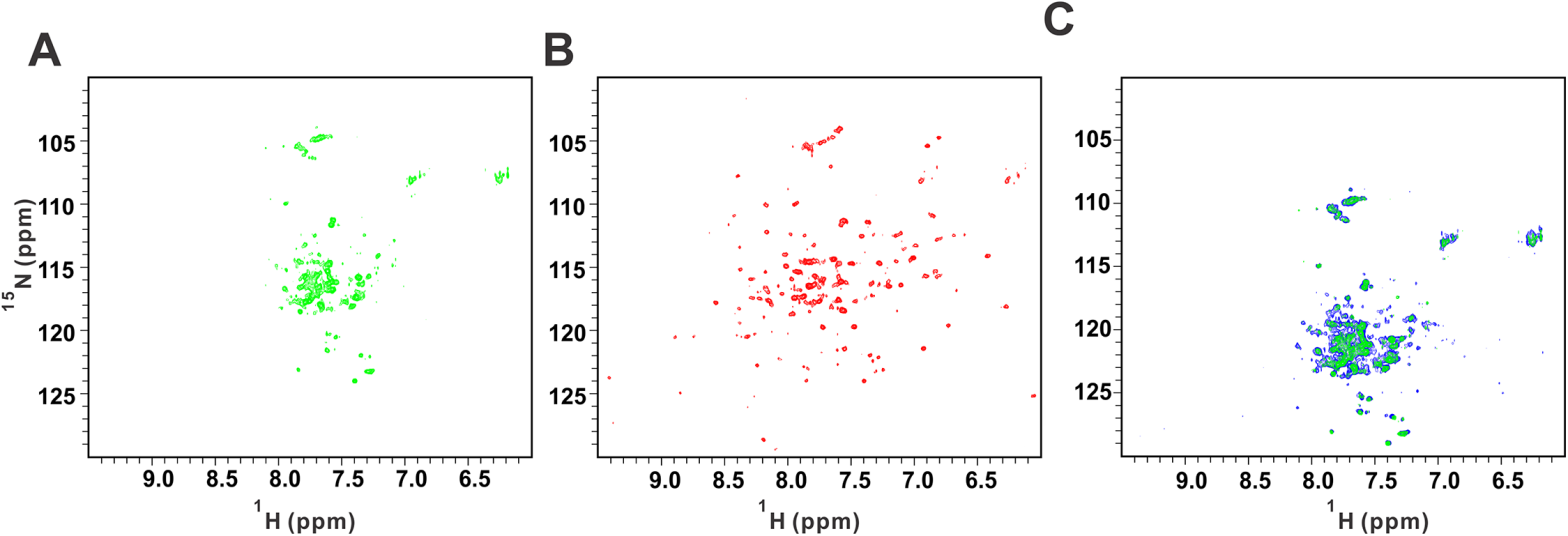
Hits validation using protein based ^1^H-^15^N HSQC. (A) Spectrum of full length HuR (green) (B) Spectrum of full length HuR with Compound C11 (red) (C) Spectral overlay of free (green) and C10 bound (blue).

**Fig 4:**
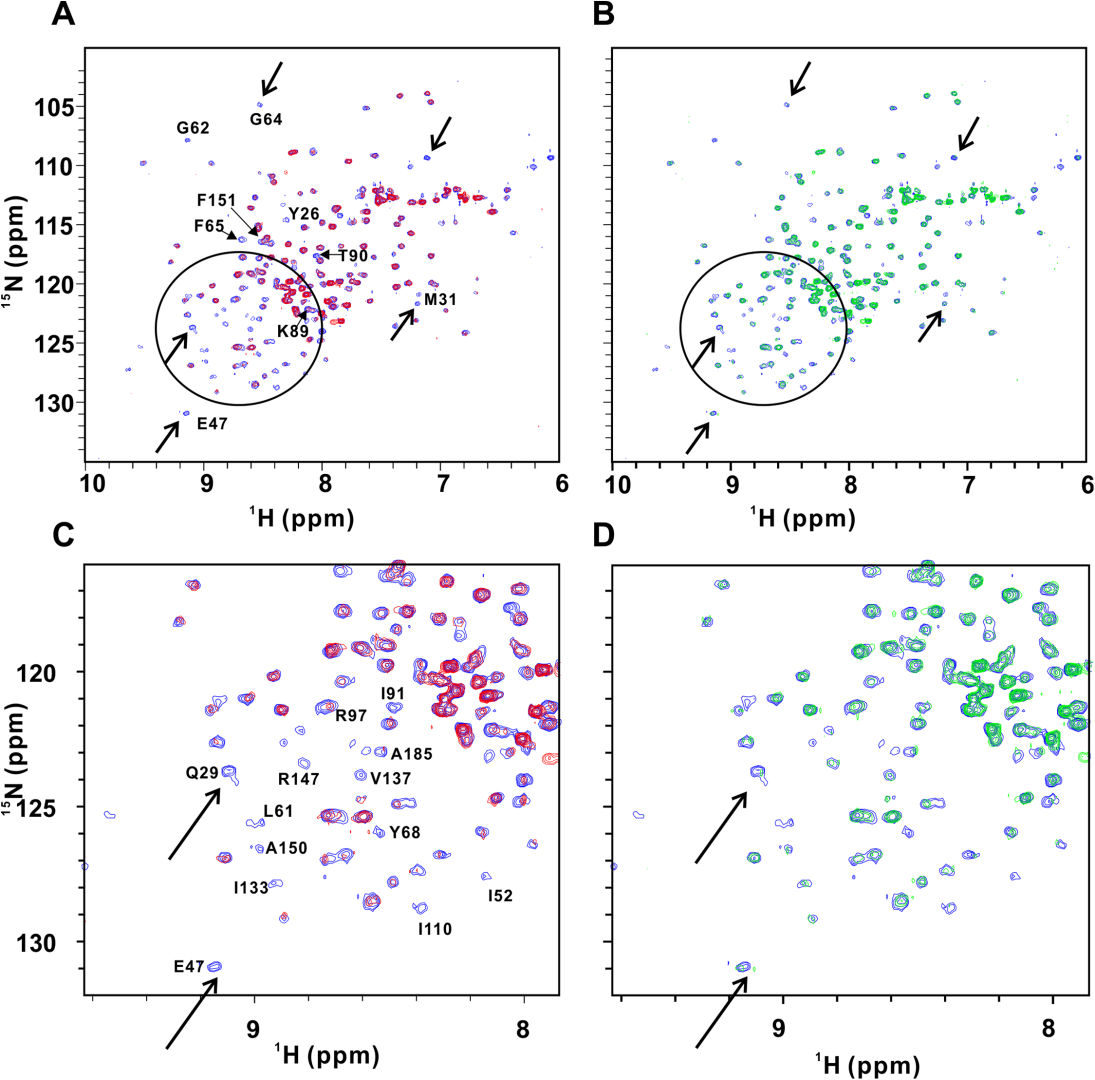
C10 displaces the RNA from RRM1-2/RNA complex. (A) Overlay of free RRM1-2 (blue) and RRM1-2/RNA (red). (B) Overlay of free RRM1-2 (blue) and RRM1-2/RNA/C10. (C) Zoom-in region of (A). (D) Zoom-in region of (B). When RNA binds to RRM1-2, many peaks disappeared as indicated in the cycle and arrow. The peaks come back once adding C10 to RRM1-2/RNA complex, indicating that C10 does block the RRM1-2/RNA interaction.

To further elucidate the mode of action of the 4 compounds selected in the secondary assay, we performed protein-detected NMR studies of their interactions with HuR. Chemical shift perturbation (CSP) is one of most robust, reliable and reproducible binding assays used today [45]: CSP is a guide for identifying the interaction sites and is very sensitive to structural changes and can be measured accurately. In the meanwhile, CSP can directly provide both an affinity measurement and a binding site using the same set of experiments. HuR is a 36KDa protein, which consists of three independently folded domains RRM1, 2 and 3, each approximately 80 amino acids long, separated by flexible linkers. Recently, it was shown that RRM3 did not interact with RRM1 or RRM2, while it can self-associate to form dimer/oligomer [49]. Our NMR titration data show that RRM1 does not interact with RRM2 either (S4 Fig). Thus, dimer/oligomer only forms via RRM3 domain. The^1^H-^15^N HSQC spectrum of the ^15^N-labeled full length protein reveals that very few signals are visible and they display poor spectral dispersion (Fig 3A). This indicates that only the flexible portions of the protein are observed whereas the folded domains broadened beyond detection because of oligomerization. Interestingly, addition of compound C11 at ratio of 2:1 (C11:HuR) results in a dramatic change in the HSQC spectrum. A set of well-dispersed weaker signals appears in the spectrum, indicating that compound C11 disrupts protein oligomerization and thus improves the quality of protein NMR spectra (Fig 3B). In the meanwhile, our AUC data show that C11 affects the formation of dimer/oligomer (S5 Fig). This is a clear indication that compound C11 directly interacts with the protein. In contrast, other compounds including compound C10 did not have a significant effect on the appearance of the HSQC spectrum of the full-length HuR (Fig 3C), although C10 affects the formation of dimer/oligomer of HuR (S5 Fig). We then investigated effect of the compounds on the ^1^H-^15^NHSQC spectra of a shorter HuR construct containing only domains RRM1-2. Only compound C10 was observed to have an effect on the spectrum of RRM1-2 (Fig 4A and 4C). Upon addition of unlabeled 11-mer single stranded RNA oligo to HuR RRM1-2, many peaks disappeared due to NMR line broadening (Fig 4A and 4C). The crystal structure of HuR RRM1-2 has been determined and protein-RNA interface was well defined [50]. To investigate HuR/RNA interaction in solution, we carried out backbone assignment for HuR RRM1-2. Due to the difficulty to obtain higher concentrated sample for ^13^C/^15^N labeled RRM1-2 (maximum concentration we can get is about 0.15mM due to the precipitation above 0.15mM), the assignment of RRM1-2 becomes more challenging task. At the end, we have managed to obtain 85% backbone assignment (S6 Fig). The chemical shifts were deposited in BMRB (accession number: 26628). We found that those peaks were indeed located at the HuR/RNA interface (Fig 4 and S7 Fig), which were consistent with those observed in crystal structure [50], although there are some residues were not fully assigned. Interestingly, once we added stoichiometric amount of compound C10 to HuR/RNA complex, most of the NMR signals broadened by interaction with RNA were restored (Fig 4B and 4D), indicating that C10 interferes with HuR/RNA binding. To investigate if there are some hits missing using STD followed by 2D HSQC, we carried out protein-based method using ^1^H-^15^N HSQC (S8 and S9 Figs). The data indicated only C10 restored the peaks which disappeared from the spectrum of HuR/RNA complex, other compounds did not.

## Discussion

RNA binding proteins (RBPs) that associate with specific mRNA and function as mRNA turnover and translation regulatory RNA binding protein (TTR-RBP) have emerged as pivotal post-transcriptional regulators of gene expression in mammalian cells [2–4]. The aberrant overexpression of RBPs in cancer led to the hypothesis that RBPs might play a pivotal role in the development of malignancies. Elevated expression of the human antigen R (HuR), for example, has been linked to carcinogenesis in many human tumors. Elevated HuR levels correlate with poor outcome and with resistance to chemotherapy [25–29]. HuR is known to stabilize mRNAs of proto-oncogenes, transcription factors, cytokines and growth factors, such as Bcl-2, SIRT1, c-Fos, COX-2, TGF-β, VEGF, TSP1 by recognizing AU-rich elements (AREs) presented in the 3’ or 5’ untranslated regions (UTRs) and promoting their expression, thus contributing to the cancerous phenotype[5–17]. HuR inhibition, therefore, may hold promise for treatment of cancers known to depend on the elevated expression of these oncogenes.

HuR contains three distinct modules known as RNA recognition motifs (RRMs): the N-terminal tandem RRM1-2 and the C-terminal RRM3 [18]. RRM1 and RRM2 are mainly responsible for binding to AREs [21,22]. RRM3 is an oligomerization domain required for cooperative assembly of HuR on the ARE substrates that are at least 18 nucleotides in length [24,49]. RRM3 did not interact with RRM1 or RRM2 [49]. In the meanwhile, RRM1 did not interact with RRM2 either (S4 Fig). These facts indicate that there is no domain-domain interaction among three RRMs. Thus, HuR inhibitors can only block dimer/oligomer formation, which has been tested using AUC experiment (S5 Fig). We propose that inhibition of HuR/RNA interaction can be achieved by distinct mechanisms: a) by directly inhibiting HuR/RNA interaction; b) attenuating HuR/RNA interaction by blocking formation of HuR oligomer; c) by blocking HuR/RNA interaction and dimerization simultaneously.

Previous efforts to discover inhibitors of HuR/RNA interaction yielded a low molecular weight inhibitor using confocal fluctuation spectroscopic assay [51], in which short construct was used for screening and there is no secondary screening included. A distinct screening strategy using the Alpha Screen method has recently been described using HuR expressed in mammalian cell-based system [52], in which full length HuR was used for screening, and EMSA as secondary screening was included. However, using EMSA for hits verification may become time-consuming and cumbersome if larger compound library used in screening (for example, 500K compounds library). Here, we set out to develop an alternative strategy using a fluorescence polarization readout that has proven to be very robust in the high throughput setting [35]. We demonstrate here that a fluorescence polarization assay performs very well with the recombinant full-length HuR produced using an *E*. *coli* expression system and a fluorescently-labeled RNA oligo. We also show that NMR-based assays can be used as a secondary filter to remove false positives arising in the primary screening assay by selecting compounds that directly interact with the target protein. During the time of preparing manuscripts, a paper using FP to screen HuR inhibitor was published [53]. SPR was used to detect direct binding, but it may still be a time-consuming step during the hits verification. Although we still use FP as primary HTS, our system is unique. First of all, NMR based method has been developed as a high throughput method compatible to conventional HTS [40] and is now used as orthogonal read-out to the biochemical HTS [41,42]. NMR based approach has been proven to be an efficient method for drug discovery [36–39]. Thus, we take advantage of modern NMR techniques and use ligand-based and protein-based approaches as orthogonal methods to HTS to remove false positive hits from FP-HTS. Second, NMR-based approach provides structure activity relationship (SAR) of inhibitors and HuR, which can be used for hit optimization. Saturation transfer difference (STD-NMR) and chemical shift perturbation using 2D ^1^H-^15^N HSQC are commonly used for studying the protein-ligand binding, which provide the information for residue specific interaction as illustrated in Fig 4. Third, our protocol will be fast-performed, due to the nature of high throughput screening of both FP and NMR in the primary and secondary screening. Finally, NMR-STD and HSQC can be used in parallel and can also be used as an approach for the cross-validation between STD and HSQC experiments (Fig 2, S8 and S9 Figs) to remove false positives. We also added AUC experiment at the end of secondary screening (S5 Fig), since AUC was commonly used as a tool for molecular interaction [54].

The primary assay yielded a ~0.75% hit rate in the test screen of the NCI diversity set V library. This hit rate makes an NMR-based secondary assay practical. STD-NMR experiments were used as the secondary screening assay using a 500 MHz NMR spectrometer equipped with an automated sample changer and a 1.7 mm cryogenic NMR probe. The 1.7 mm probe requires less than 40 uL of sample volume, thus less than 10 micrograms of compound per sample was used in the secondary STD-NMR assay. Protein concentration in the STD-NMR samples was 10 uM, making protein consumption in the secondary assay also very low. Once the hit pool was further narrowed down by identifying compounds that directly interact with the HuR protein, we used protein-detected NMR experiments to further elucidate the mode of action of the STD-NMR positive compounds. Protein-detected NMR experiments require larger amounts of isotopically labeled protein, thus their utility in the screening efforts is limited by their modest throughput, particularly for larger molecular weight proteins. Nevertheless, they are still ideal as the last validation step of the already limited pool of candidate compounds, because of their sensitivity to even relatively week protein/small molecule interactions and their ability to map the interaction site. Furthermore, ^1^H-^15^N HSQC experiments can be used to evaluate effects of the compounds on protein oligomerization and protein/RNA interaction as is demonstrated in this study. HSQC spectra revealed that compound C11 restores signals that are not observed in the spectra of the full-length HuR protein. This striking effect most likely arises from the inhibition of HuR oligomerization caused by C11 binding to the protein, since there is no domain-domain interaction among three RRMs ([49], S4 Fig). In contrast, compound C10 has no or little effect on the spectra of the full-length protein, but its effect on the RNA binding is revealed in the HSQC spectra collected on the shorter RRM1-2 construct. These results illustrate the power of the protein-detected HSQC experiments for elucidation of the inhibitory mechanism of the small molecule ligands. The utility of the HSQC spectra in compound screening and characterization may be further improved if individual domain constructs are used to detect interaction with the hit compounds. These efforts are currently in progress.

In summary, our preliminary studies reveal that small molecules can inhibit HuR/RNA interaction either by directly competing for the RNA binding site on the RRM1-2 segment (Fig 4) or indirectly interfering with HuR oligomerization mediated by the RRM3 module (Fig 3B). Performance of our screening strategy on the NCI diversity set V library suggests that the method is sufficiently robust for screening of larger compound libraries.

## Supporting Information

S1 Fig HTS screening using NCI diversity set V compounds library. (A) The standard deviation is 18.2 in unfiltered raw data. The average FP value is 171.22 (red solid line). Compounds producing FP readings significantly higher than the average (2σ, 2x18.2, blue line) were ignored due to protein precipitation and/or fluorescence quenching caused by compounds. The hits having 5-standard deviation (5x18.2, cyan line) below the average were also removed due to their intrinsic fluorescence. (B) The standard deviation was 12.4 with the average of fluorescence reading at 171.40 (red dotted line) after the removal of 18 compounds from (A). The hits having 3-standard deviation (3x12.4, green line) below the average were selected for further verification. (C) The selected hits from (B) were subjected retesting using original condition. The cut-off is same as initial hits pick-up.
(TIF)

S2 Fig Dose responses of hits on confirmed hits from primary screening. Compounds at 250uM in final concentration in the assay volume of 25uL were added to the mixture of 10nM RNA and 50nM HuR in the first well in 384 format plate followed by a serial dilution into next well where the concentration of RNA and HuR was kept constant.
(TIF)

S3 Fig Chemical structures of selected hits. (TIF)

S4 Fig The spectral overlay between RRM1 and mixture of RRM1 with unlabeled RRM2. Unlabeled RRM2 was titrated into 0.1mM ^15^N-labeled RRM1. The end-point spectrum (RRM2/RRM1 = 9:1) (red) was overlaid with that of free RRM1 (blue). Two spectra overlaid very well, indicating that RRM1 did not interact with RRM2.
(TIF)

S5 Fig Post-hit validation using analytical ultracentrifugation (AUC). Sedimentation velocity studies are informative of the overall shape of molecule. The predicted moomer of full length HuR (36 kDa) is ~2.5x10^13^ (vertical light blue line) using Ultrascan III software, while our AUC data indicated that only ~10% monomer existed in solution, the majority of species was dimer/oligomer. We tested 4 compounds from secondary screening. 30% and 50% fraction of boundary were in orange. 10uM HuR and 20uM compounds were used in AUC experiments in the total volume of 500ul. We found that both C10 and C11 shifted the boundary of species toward monomer, while C2 and C8 did not, indicating that C10 and C11 partially disrupted the formation of HuR dimer/oligomer.
(TIF)

S6 Fig Backbone assignment of HuR RRM1-2 (residues 1–186). (TIF)

S7 Fig Chemical shift mapping of HuR/RNA interface. The cartoon representation of crystal structure of HuR RRM1-2 was shown. RRM1 and RRM2 are colored green. RNA is in orange. The residues interact with RNA was shown in sticks and colored red.
(TIF)

S8 Fig The spectral overlay of HuR/RNA complex with or without compounds. The panel A-F represents compounds C1-6 respectively.
(TIF)

S9 Fig The spectral overlay of HuR/RNA complex with or without compounds. The panel A-F represents compounds C7-12 respectively.
(TIF)

S1 Table IC50 for confirmed hits from primary HTS screening. IC50s were obtained by fitting to the equation of ΔP = ΔPmax/(1+10^((Logx_0_-X)*p)).
(DOC)

## References

[1] Audic Y, Hartley RS (2004) Post-transcriptional regulation in cancer. Biol Cell 96: 479–498. 15380615 10.1016/j.biolcel.2004.05.002

[2] Glisovic T, Bachorik JL, Yong J, Dreyfuss G (2008) RNA-binding proteins and post-transcriptional gene regulation. FEBS Lett 582: 1977–1986. 10.1016/j.febslet.2008.03.004 18342629 PMC2858862

[3] Derrigo M, Cestelli A, Savettieri G, Di Liegro I (2000) RNA-protein interactions in the control of stability and localization of messenger RNA (review). Int J Mol Med 5: 111–123. 10639588

[4] Pullmann R Jr., Kim HH, Abdelmohsen K, Lal A, Martindale JL, et al (2007) Analysis of turnover and translation regulatory RNA-binding protein expression through binding to cognate mRNAs. Mol Cell Biol 27: 6265–6278. 17620417 10.1128/MCB.00500-07 PMC2099612

[5] Chen CY, Shyu AB (1995) AU-rich elements: characterization and importance in mRNA degradation. Trends Biochem Sci 20: 465–470. 8578590 10.1016/s0968-0004(00)89102-1

[6] Schiavi SC, Belasco JG, Greenberg ME (1992) Regulation of proto-oncogene mRNA stability. Biochimica et Biophysica Acta (BBA)—Reviews on Cancer 1114: 95–106. 1457466 10.1016/0304-419x(92)90009-n

[7] Barreau C, Paillard L, Osborne HB (2005) AU-rich elements and associated factors: are there unifying principles? Nucleic Acids Res 33: 7138–7150. 16391004 10.1093/nar/gki1012 PMC1325018

[8] Wurth L (2012) Versatility of RNA-Binding Proteins in Cancer. Comp Funct Genomics 2012: 178525 10.1155/2012/178525 22666083 PMC3359819

[9] Abdelmohsen K, Lal A, Kim HH, Gorospe M (2007) Posttranscriptional orchestration of an anti-apoptotic program by HuR. Cell Cycle 6: 1288–1292. 17534146 10.4161/cc.6.11.4299

[10] Abdelmohsen K, Pullmann R Jr., Lal A, Kim HH, Galban S, et al (2007) Phosphorylation of HuR by Chk2 regulates SIRT1 expression. Mol Cell 25: 543–557. 17317627 10.1016/j.molcel.2007.01.011 PMC1986740

[11] Ishimaru D, Ramalingam S, Sengupta TK, Bandyopadhyay S, Dellis S, et al (2009) Regulation of Bcl-2 expression by HuR in HL60 leukemia cells and A431 carcinoma cells. Mol Cancer Res 7: 1354–1366. 10.1158/1541-7786.MCR-08-0476 19671677

[12] Akool el S, Kleinert H, Hamada FM, Abdelwahab MH, Forstermann U, et al (2003) Nitric oxide increases the decay of matrix metalloproteinase 9 mRNA by inhibiting the expression of mRNA-stabilizing factor HuR. Mol Cell Biol 23: 4901–4916. 12832476 10.1128/MCB.23.14.4901-4916.2003 PMC162218

[13] Huwiler A, Akool el S, Aschrafi A, Hamada FM, Pfeilschifter J, et al (2003) ATP potentiates interleukin-1 beta-induced MMP-9 expression in mesangial cells via recruitment of the ELAV protein HuR. J Biol Chem 278: 51758–51769. 14523003 10.1074/jbc.M305722200

[14] Tran H, Maurer F, Nagamine Y (2003) Stabilization of urokinase and urokinase receptor mRNAs by HuR is linked to its cytoplasmic accumulation induced by activated mitogen-activated protein kinase-activated protein kinase 2. Mol Cell Biol 23: 7177–7188. 14517288 10.1128/MCB.23.20.7177-7188.2003 PMC230330

[15] Dong R, Lu JG, Wang Q, He XL, Chu YK, et al (2007) Stabilization of Snail by HuR in the process of hydrogen peroxide induced cell migration. Biochem Biophys Res Commun 356: 318–321. 17350594 10.1016/j.bbrc.2007.02.145

[16] Filippova N, Yang X, Wang Y, Gillespie GY, Langford C, et al (2011) The RNA-binding protein HuR promotes glioma growth and treatment resistance. Mol Cancer Res 9: 648–659. 10.1158/1541-7786.MCR-10-0325 21498545 PMC3096748

[17] Wang W, Caldwell MC, Lin S, Furneaux H, Gorospe M (2000) HuR regulates cyclin A and cyclin B1 mRNA stability during cell proliferation. EMBO J 19: 2340–2350. 10811625 10.1093/emboj/19.10.2340 PMC384372

[18] Ma WJ, Cheng S, Campbell C, Wright A, Furneaux H (1996) Cloning and characterization of HuR, a ubiquitously expressed Elav-like protein. J Biol Chem 271: 8144–8151. 8626503 10.1074/jbc.271.14.8144

[19] Maris C, Dominguez C, Allain FH (2005) The RNA recognition motif, a plastic RNA-binding platform to regulate post-transcriptional gene expression. FEBS J 272: 2118–2131. 15853797 10.1111/j.1742-4658.2005.04653.x

[20] Scheiba RM, Aroca A, Diaz-Moreno I (2012) HuR thermal stability is dependent on domain binding and upon phosphorylation. Eur Biophys J 41: 597–605. 10.1007/s00249-012-0827-3 22706953

[21] Fan XC, Steitz JA (1998) Overexpression of HuR, a nuclear-cytoplasmic shuttling protein, increases the in vivo stability of ARE-containing mRNAs. EMBO J 17: 3448–3460. 9628880 10.1093/emboj/17.12.3448 PMC1170681

[22] Uren PJ, Burns SC, Ruan J, Singh KK, Smith AD, et al (2011) Genomic analyses of the RNA-binding protein Hu antigen R (HuR) identify a complex network of target genes and novel characteristics of its binding sites. J Biol Chem 286: 37063–37066. 10.1074/jbc.C111.266882 21890634 PMC3199453

[23] Ma WJ, Chung S, Furneaux H (1997) The Elav-like proteins bind to AU-rich elements and to the poly(A) tail of mRNA. Nucleic Acids Res 25: 3564–3569. 9278474 10.1093/nar/25.18.3564 PMC146929

[24] Fialcowitz-White EJ, Brewer BY, Ballin JD, Willis CD, Toth EA, et al (2007) Specific protein domains mediate cooperative assembly of HuR oligomers on AU-rich mRNA-destabilizing sequences. J Biol Chem 282: 20948–20959. 17517897 10.1074/jbc.M701751200 PMC2244793

[25] Erkinheimo TL, Lassus H, Sivula A, Sengupta S, Furneaux H, et al (2003) Cytoplasmic HuR expression correlates with poor outcome and with cyclooxygenase 2 expression in serous ovarian carcinoma. Cancer Res 63: 7591–7594. 14633672

[26] Zhu Z, Wang B, Bi J, Zhang C, Guo Y, et al (2013) Cytoplasmic HuR expression correlates with P-gp, HER-2 positivity, and poor outcome in breast cancer. Tumour Biol. 10.1007/s13277-013-0774-3 23605320

[27] Liang PI, Li WM, Wang YH, Wu TF, Wu WR, et al (2012) HuR cytoplasmic expression is associated with increased cyclin A expression and poor outcome with upper urinary tract urothelial carcinoma. BMC Cancer 12: 611 10.1186/1471-2407-12-611 23259573 PMC3571926

[28] Wang J, Wang B, Bi J, Zhang C (2011) Cytoplasmic HuR expression correlates with angiogenesis, lymphangiogenesis, and poor outcome in lung cancer. Med Oncol 28 Suppl 1: S577–585. 10.1007/s12032-010-9734-6 21046284

[29] Ronkainen H, Vaarala MH, Hirvikoski P, Ristimaki A (2011) HuR expression is a marker of poor prognosis in renal cell carcinoma. Tumour Biol 32: 481–487. 10.1007/s13277-010-0141-6 21161467

[30] Nabors LB, Gillespie GY, Harkins L, King PH (2001) HuR, a RNA stability factor, is expressed in malignant brain tumors and binds to adenine- and uridine-rich elements within the 3' untranslated regions of cytokine and angiogenic factor mRNAs. Cancer Res 61: 2154–2161. 11280780

[31] Vo DT, Abdelmohsen K, Martindale JL, Qiao M, Tominaga K, et al (2012) The oncogenic RNA-binding protein Musashi1 is regulated by HuR via mRNA translation and stability in glioblastoma cells. Mol Cancer Res 10: 143–155. 10.1158/1541-7786.MCR-11-0208 22258704 PMC3265026

[32] Hostetter C, Licata LA, Witkiewicz A, Costantino CL, Yeo CJ, et al (2008) Cytoplasmic accumulation of the RNA binding protein HuR is central to tamoxifen resistance in estrogen receptor positive breast cancer cells. Cancer Biol Ther 7: 1496–1506. 18769129 10.4161/cbt.7.9.6490

[33] Yuan Z, Sanders AJ, Ye L, Wang Y, Jiang WG (2011) Knockdown of human antigen R reduces the growth and invasion of breast cancer cells in vitro and affects expression of cyclin D1 and MMP-9. Oncol Rep 26: 237–245. 10.3892/or.2011.1271 21503589

[34] Winkler C, Doller A, Imre G, Badawi A, Schmid T, et al (2014) Attenuation of the ELAV1-like protein HuR sensitizes adenocarcinoma cells to the intrinsic apoptotic pathway by increasing the translation of caspase-2L. Cell Death Dis 5: e1321 10.1038/cddis.2014.279 25010987 PMC4123073

[35] Lea WA, Simeonov A (2011) Fluorescence polarization assays in small molecule screening. Expert Opin Drug Discov 6: 17–32. 10.1517/17460441.2011.537322 22328899 PMC3277431

[36] Mortezai N, Behnken HN, Kurze AK, Ludewig P, Buck F, et al (2013) Tumor-associated Neu5Ac-Tn and Neu5Gc-Tn antigens bind to C-type lectin CLEC10A (CD301, MGL). Glycobiology 23: 844–852. 10.1093/glycob/cwt021 23507963

[37] Germer A, Mugge C, Peter MG, Rottmann A, Kleinpeter E (2003) Solution- and bound-state conformational study of N,N',N"-triacetyl chitotriose and other analogous potential inhibitors of hevamine: application of trNOESY and STD NMR spectroscopy. Chemistry 9: 1964–1973. 12740843 10.1002/chem.200204231

[38] Wu B, Zhang Z, Noberini R, Barile E, Giulianotti M, et al (2013) HTS by NMR of combinatorial libraries: a fragment-based approach to ligand discovery. Chem Biol 20: 19–33. 10.1016/j.chembiol.2012.10.015 23352136 PMC3966493

[39] Scott DE, Ehebauer MT, Pukala T, Marsh M, Blundell TL, et al (2013) Using a fragment-based approach to target protein-protein interactions. Chembiochem 14: 332–342. 10.1002/cbic.201200521 23344974 PMC3594973

[40] Dalvit C, Flocco M, Knapp S, Mostardini M, Perego R, et al (2002) High-throughput NMR-based screening with competition binding experiments. J Am Chem Soc 124: 7702–7709. 12083923 10.1021/ja020174b

[41] Evenas J, Edfeldt F, Lepisto M, Svitacheva N, Synnergren A, et al (2014) HTS followed by NMR based counterscreening. Discovery and optimization of pyrimidones as reversible and competitive inhibitors of xanthine oxidase. Bioorg Med Chem Lett 24: 1315–1321. 10.1016/j.bmcl.2014.01.050 24508129

[42] Hajduk PJ, Burns DJ (2002) Integration of NMR and high-throughput screening. Comb Chem High Throughput Screen 5: 613–621. 12470258 10.2174/1386207023329996

[43] Goldflam M, Tarrago T, Gairi M, Giralt E (2012) NMR studies of protein-ligand interactions. Methods Mol Biol 831: 233–259. 10.1007/978-1-61779-480-3_14 22167678

[44] Engsberg JR, Lenke LG, Hollander KW, Uhrich ML, Commean PK, et al (2003) Methods to locate center of gravity in scoliosis. Spine (Phila Pa 1976) 28: E483–489. 14652482 10.1097/01.BRS.0000099093.36335.16

[45] Benie AJ, Moser R, Bauml E, Blaas D, Peters T (2003) Virus-ligand interactions: identification and characterization of ligand binding by NMR spectroscopy. J Am Chem Soc 125: 14–15. 12515488 10.1021/ja027691e

[46] Kimple AJ, Yasgar A, Hughes M, Jadhav A, Willard FS, et al (2008) A high throughput fluorescence polarization assay for inhibitors of the GoLoco motif/G-alpha interaction. Comb Chem High Throughput Screen 11: 396–409. 18537560 10.2174/138620708784534770 PMC2440576

[47] Moerke NJ (2009) Fluorescence Polarization (FP) Assays for Monitoring Peptide‐Protein or Nucleic Acid‐Protein Binding. Current protocols in chemical biology: 1–15. 10.1002/9780470559277.ch090102 23839960

[48] Zhang JH, Chung TD, Oldenburg KR (1999) A Simple Statistical Parameter for Use in Evaluation and Validation of High Throughput Screening Assays. J Biomol Screen 4: 67–73. 10838414 10.1177/108705719900400206

[49] Scheiba RM, de Opakua AI, Diaz-Quintana A, Cruz-Gallardo I, Martinez-Cruz LA, et al (2014) The C-terminal RNA binding motif of HuR is a multi-functional domain leading to HuR oligomerization and binding to U-rich RNA targets. RNA Biol 11: 1250–1261. 10.1080/15476286.2014.996069 25584704 PMC4615805

[50] Wang H, Zeng F, Liu Q, Liu H, Liu Z, et al (2013) The structure of the ARE-binding domains of Hu antigen R (HuR) undergoes conformational changes during RNA binding. Acta Crystallogr D Biol Crystallogr 69: 373–380. 10.1107/S0907444912047828 23519412

[51] Meisner NC, Hintersteiner M, Mueller K, Bauer R, Seifert JM, et al (2007) Identification and mechanistic characterization of low-molecular-weight inhibitors for HuR. Nat Chem Biol 3: 508–515. 17632515 10.1038/nchembio.2007.14

[52] D'Agostino VG, Adami V, Provenzani A (2013) A novel high throughput biochemical assay to evaluate the HuR protein-RNA complex formation. PLoS One 8: e72426 10.1371/journal.pone.0072426 23951323 PMC3741180

[53] Wu X, Lan L, Wilson DM, Marquez RT, Tsao WC, et al (2015) Identification and validation of novel small molecule disruptors of HuR-mRNA interaction. ACS Chem Biol 10: 1476–1484. 10.1021/cb500851u 25750985 PMC4631057

[54] Cole JL, Lary JW, Moody T, Laue TM (2008) Analytical Ultracentrifugation: Sedimentation Velocity and Sedimentation Equilibrium. Methods in cell biology 84: 143–179. 17964931 10.1016/S0091-679X(07)84006-4 PMC2711687

