# Supplementary figures and images for: Identification of Small-Molecule Inhibitors of the HuR/RNA Interaction Using a Fluorescence Polarization Screening Assay Followed by NMR Validation
Source: PLoS One. 2015 Sep 21;10(9):e0138780. doi: 10.1371/journal.pone.0138780 (PMC4577092; doi:10.1371/journal.pone.0138780)

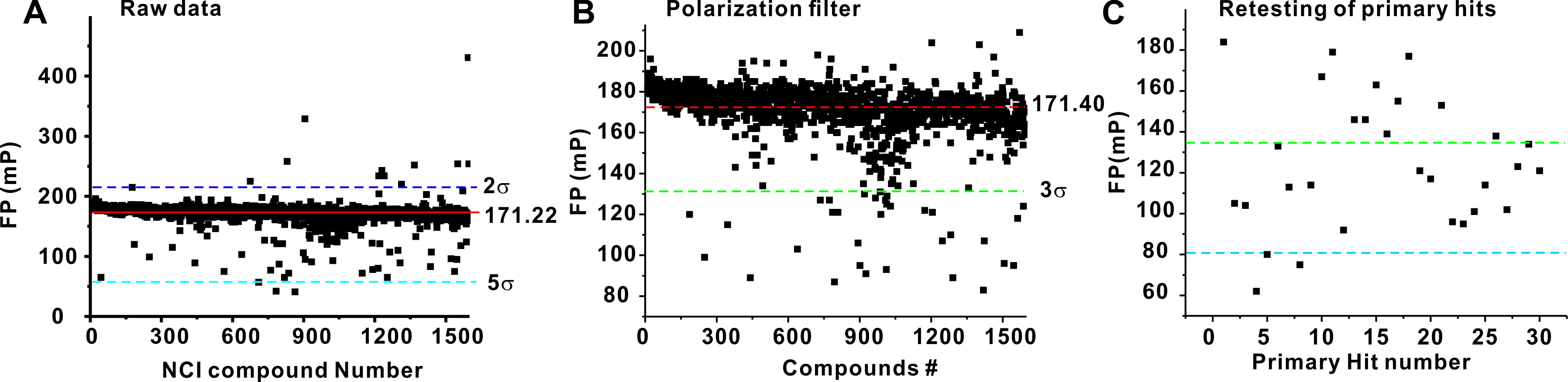

Supplement: S1 Fig — (A) The standard deviation is 18.2 in unfiltered raw data. The average FP value is 171.22 (red solid line). Compounds producing FP readings significantly higher than the average (2σ, 2x18.2, blue line) were ignored due to protein precipitation and/or fluorescence quenching caused by compounds. The hits having 5-standard deviation (5x18.2, cyan line) below the average were also removed due to their intrinsic fluorescence. (B) The standard deviation was 12.4 with the average of fluorescence reading at 171.40 (red dotted line) after the removal of 18 compounds from (A). The hits having 3-standard deviation (3x12.4, green line) below the average were selected for further verification. (C) The selected hits from (B) were subjected retesting using original condition. The cut-off is same as initial hits pick-up. (TIF) [file pone.0138780.s001.tif]

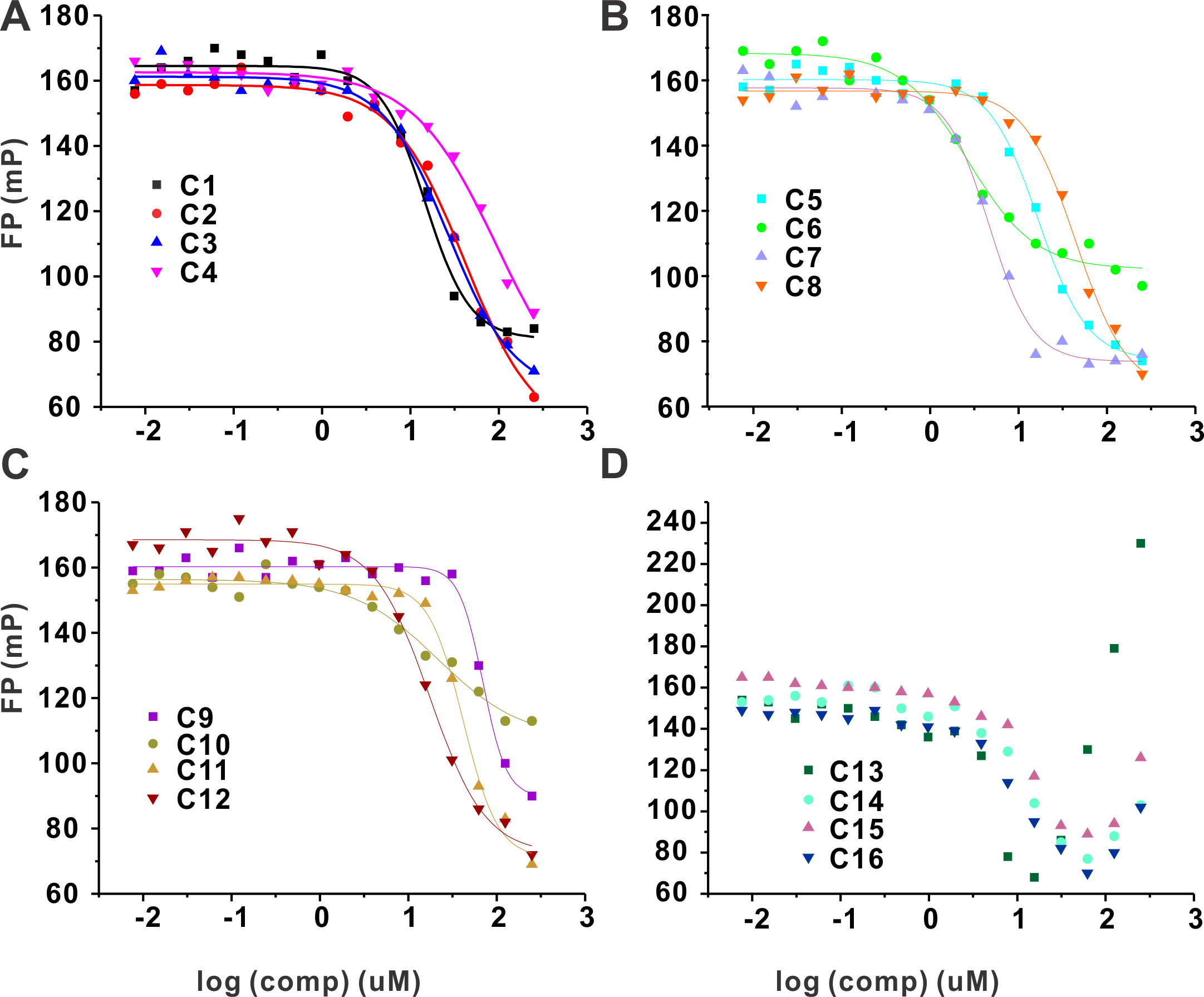

Supplement: S2 Fig — Compounds at 250uM in final concentration in the assay volume of 25uL were added to the mixture of 10nM RNA and 50nM HuR in the first well in 384 format plate followed by a serial dilution into next well where the concentration of RNA and HuR was kept constant. (TIF) [file pone.0138780.s002.tif]

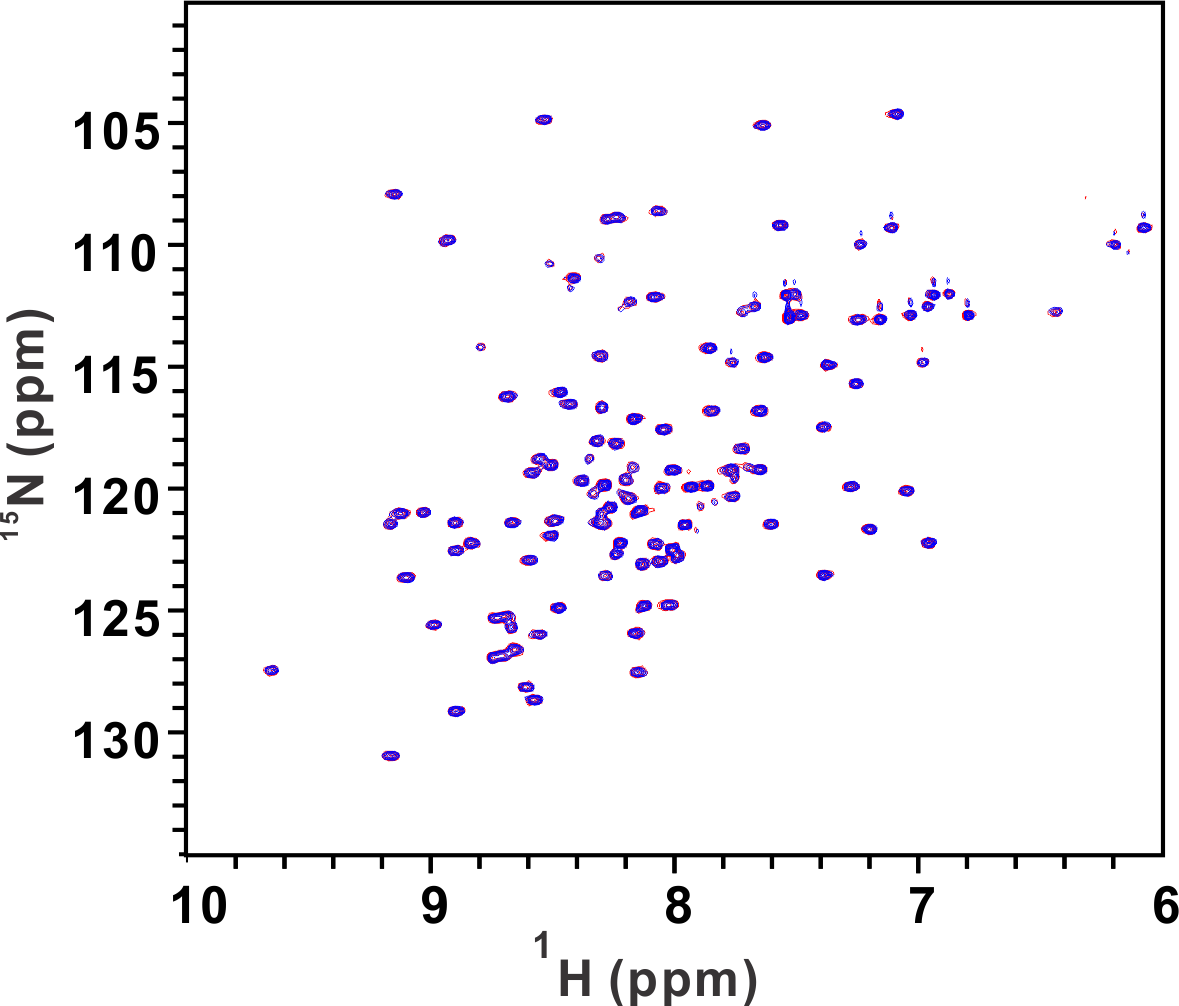

Supplement: S4 Fig — Unlabeled RRM2 was titrated into 0.1mM 15N-labeled RRM1. The end-point spectrum (RRM2/RRM1 = 9:1) (red) was overlaid with that of free RRM1 (blue). Two spectra overlaid very well, indicating that RRM1 did not interact with RRM2. (TIF) [file pone.0138780.s004.tif]

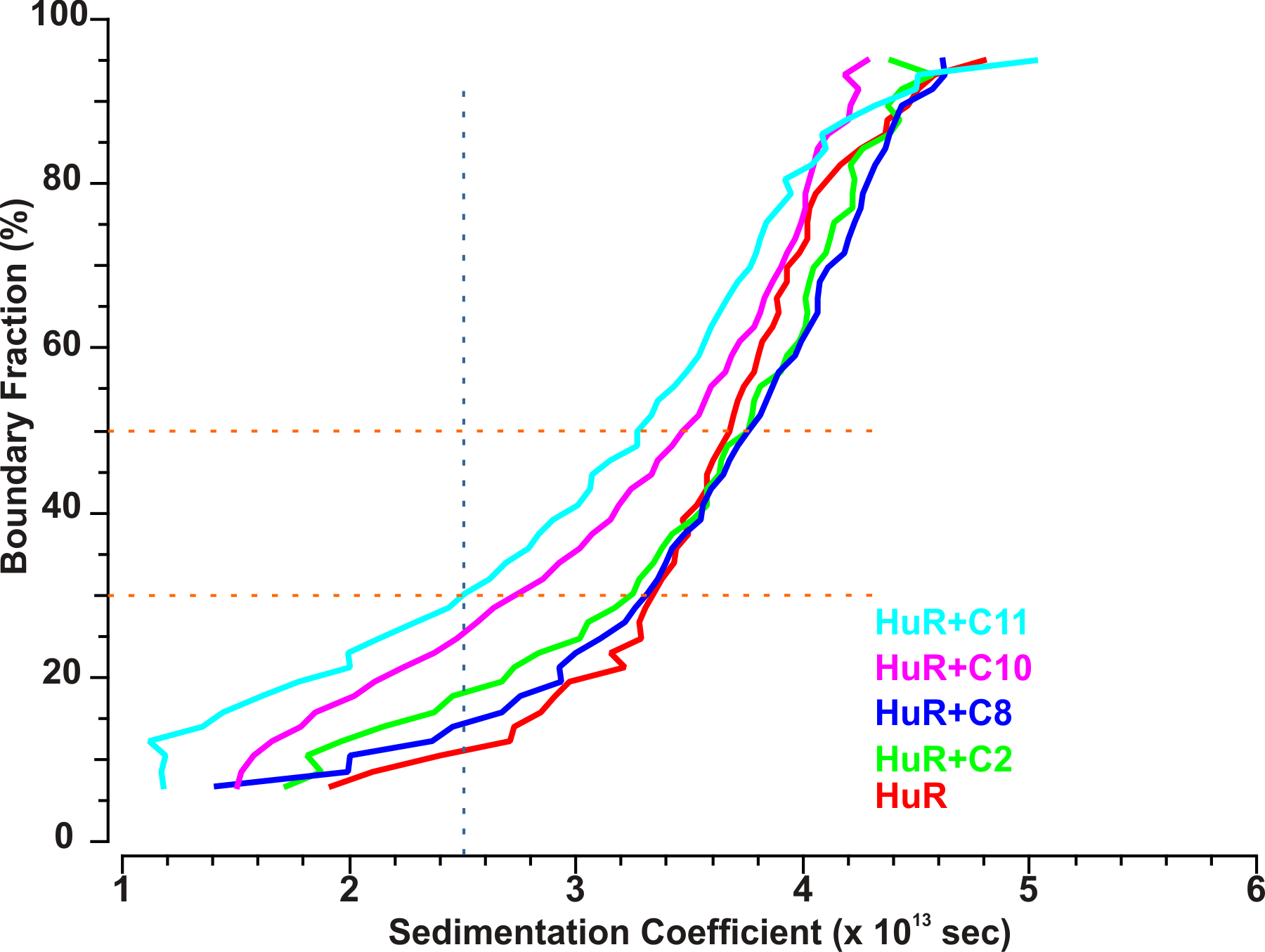

Supplement: S5 Fig — Sedimentation velocity studies are informative of the overall shape of molecule. The predicted moomer of full length HuR (36 kDa) is ~2.5x10^13^ (vertical light blue line) using Ultrascan III software, while our AUC data indicated that only ~10% monomer existed in solution, the majority of species was dimer/oligomer. We tested 4 compounds from secondary screening. 30% and 50% fraction of boundary were in orange. 10uM HuR and 20uM compounds were used in AUC experiments in the total volume of 500ul. We found that both C10 and C11 shifted the boundary of species toward monomer, while C2 and C8 did not, indicating that C10 and C11 partially disrupted the formation of HuR dimer/oligomer. (TIF) [file pone.0138780.s005.tif]

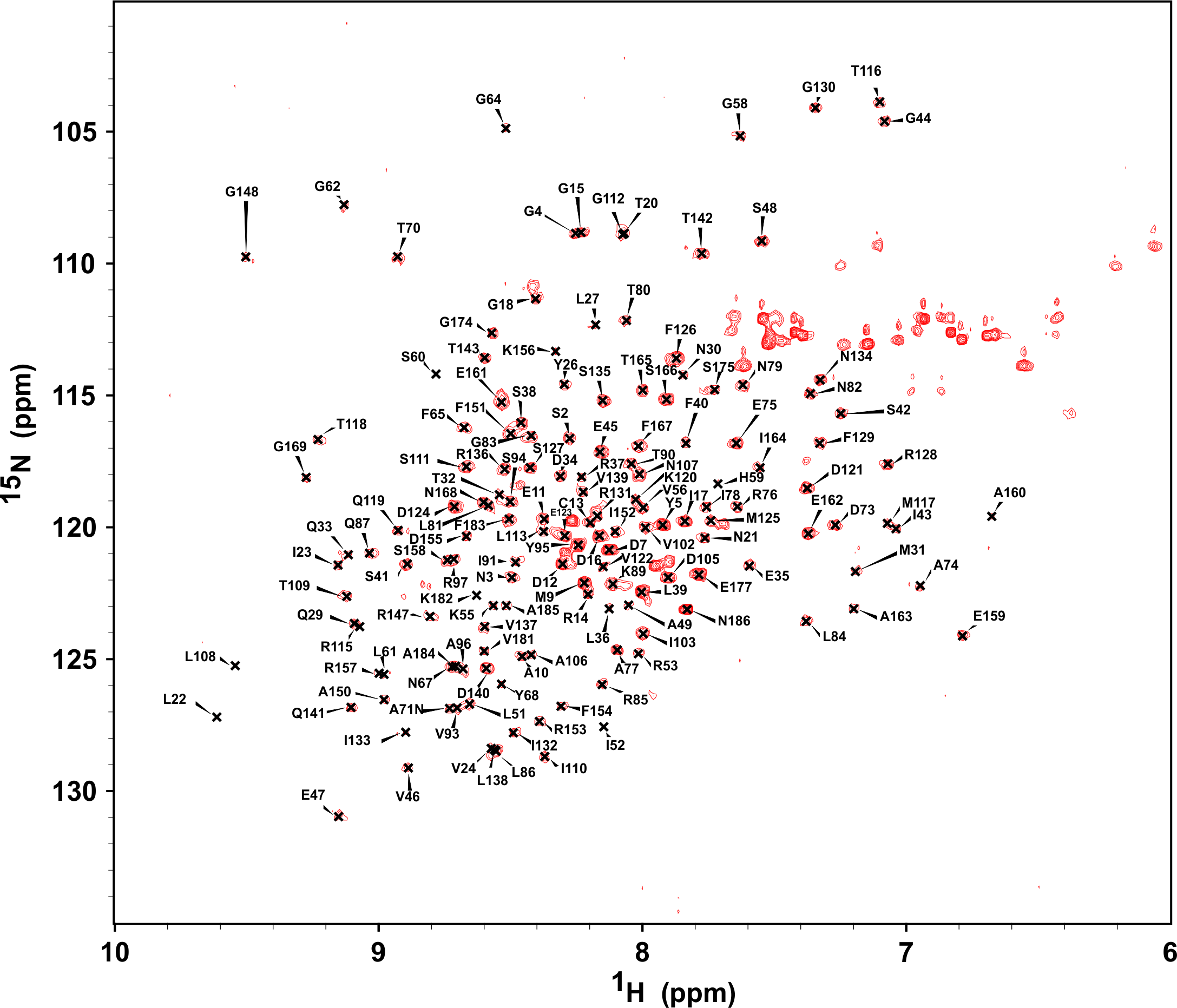

Supplement: S6 Fig — (TIF) [file pone.0138780.s006.tif]

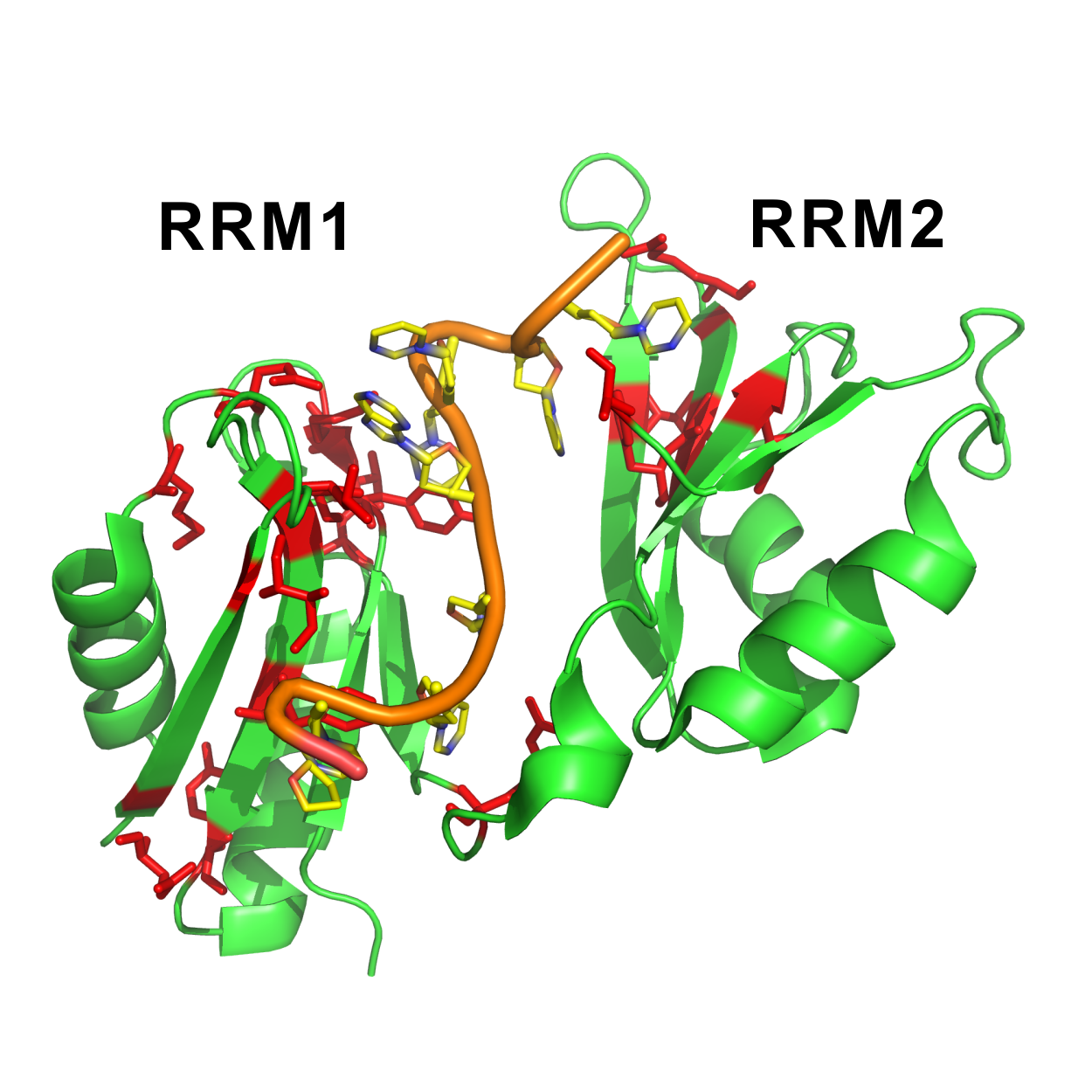

Supplement: S7 Fig — RRM1 and RRM2 are colored green. RNA is in orange. The residues interact with RNA was shown in sticks and colored red. (TIF) [file pone.0138780.s007.tif]

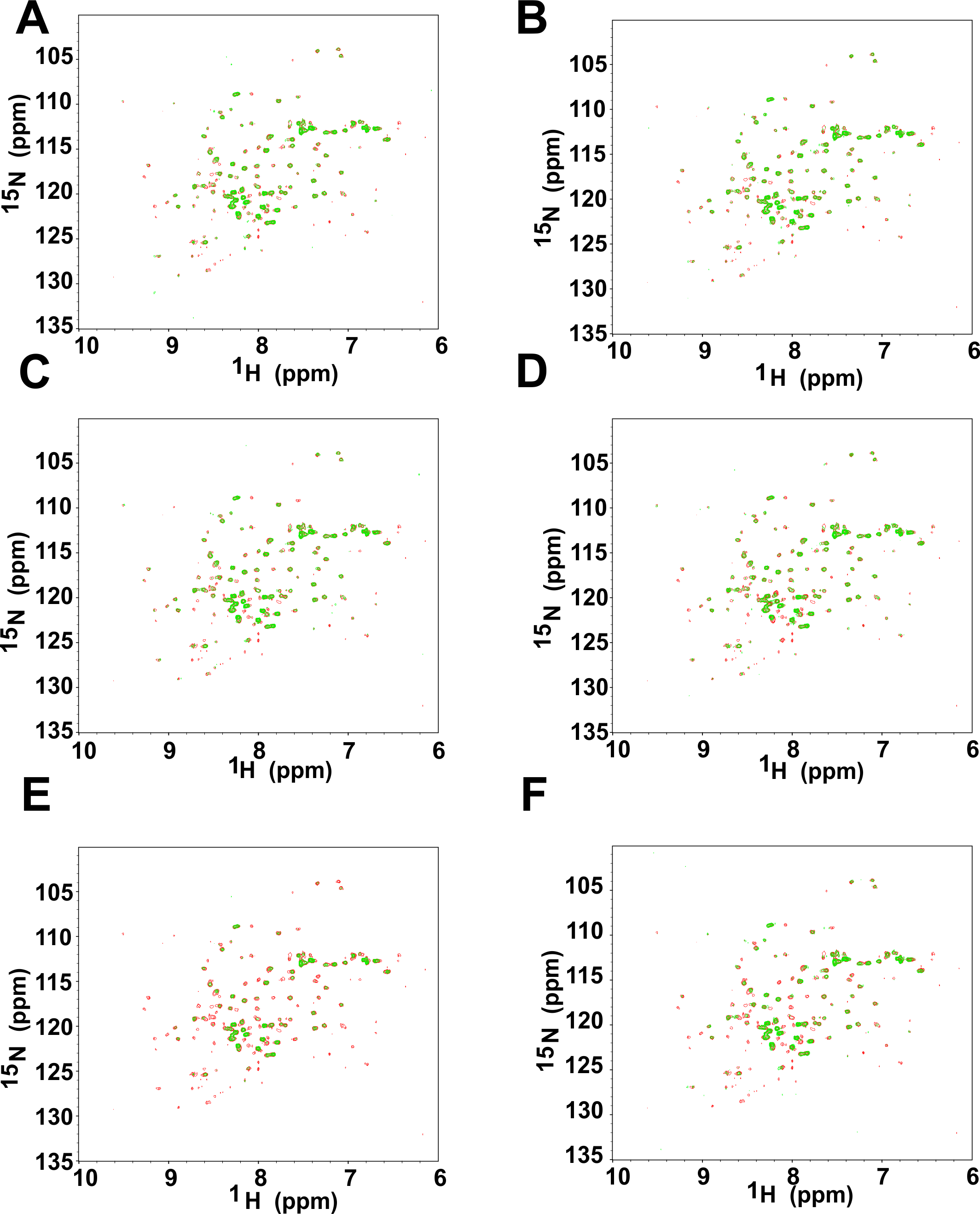

Supplement: S8 Fig — The panel A-F represents compounds C1-6 respectively. (TIF) [file pone.0138780.s008.tif]

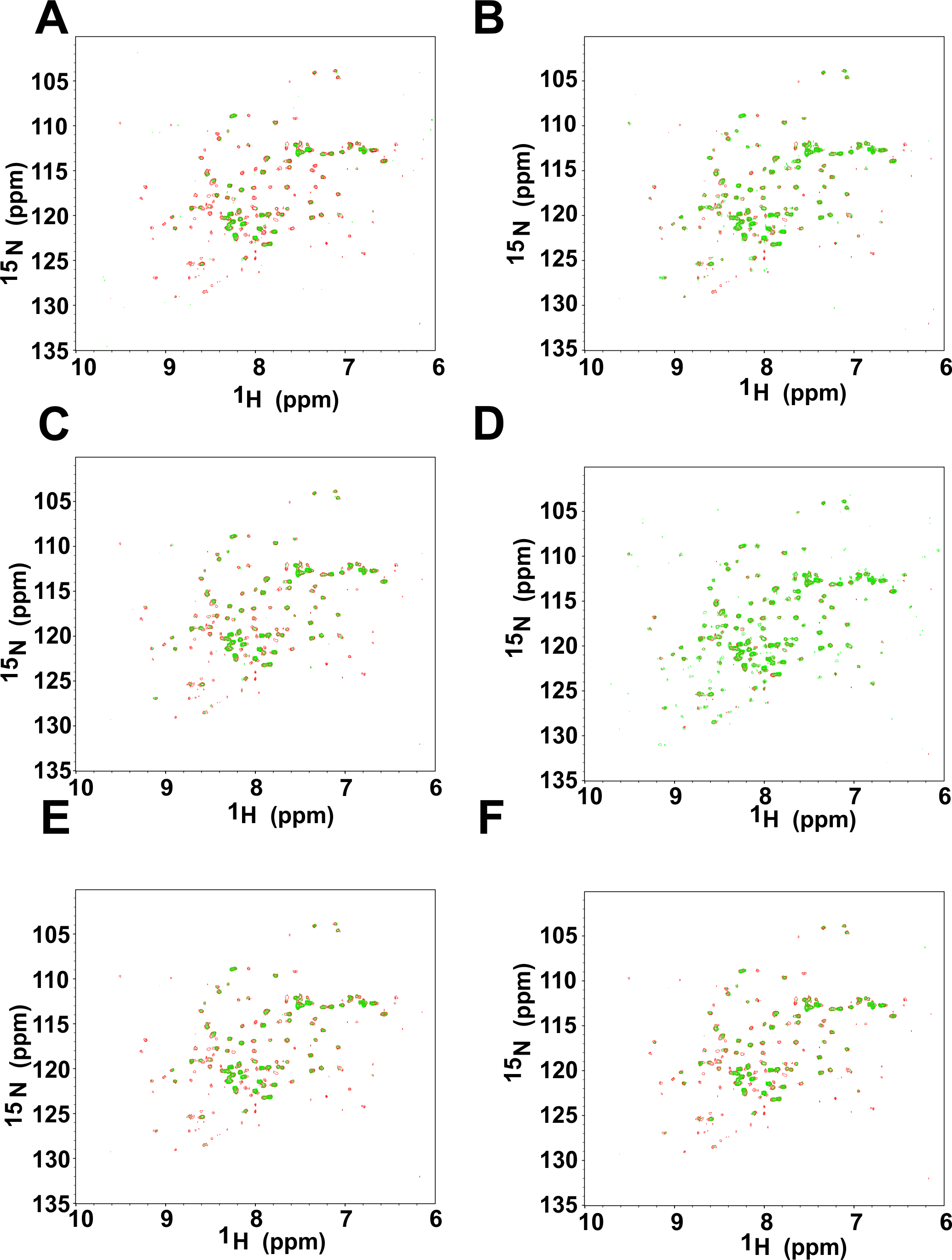

Supplement: S9 Fig — The panel A-F represents compounds C7-12 respectively. (TIF) [file pone.0138780.s009.tif]
