# Supplementary material for: Identification of Small-Molecule Inhibitors of the HuR/RNA Interaction Using a Fluorescence Polarization Screening Assay Followed by NMR Validation
Source: PLoS One. 2015 Sep 21;10(9):e0138780. doi: 10.1371/journal.pone.0138780 (PMC4577092; doi:10.1371/journal.pone.0138780)
Supplement: S1 Table — IC50s were obtained by fitting to the equation of ΔP = ΔPmax/(1+10^((Logx0-X)*p)). (DOC) [file pone.0138780.s010.doc]

| Table 1. IC50 for 12 compounds from primary HTS screening | | | | | |
| --- | --- | --- | --- | --- | --- |
| Comp# | NSC# | IC50(uM) | Comp# | NSC# | IC50(uM) |
| C1 | 5836 | 14.7 | C7 | 91438 | 4.6 |
| C2 | 7572 | 41.0 | C8 | 109292 | 44.4 |
| C3 | 44750 | 26.9 | C9 | 123418 | 69.6 |
| C4 | 50648 | 97.4 | C10 | 143491 | 21.7 |
| C5 | 62685 | 16.7 | C11 | 227186 | 41.9 |
| C6 | 84126 | 2.7 | C12 | 651084 | 17.4 |
